# Item analysis of the KIDSCREEN-10 using Rasch modelling

**DOI:** 10.1186/s12955-020-01596-6

**Published:** 2020-10-15

**Authors:** Marianne Müller, Andrea Haenni Hoti

**Affiliations:** 1 University of Teachers Education, Lucerne, Switzerland; 2 School of Engineering, Zurich University of Applied Sciences, Rosenstr. 3, 8400 Winterthur, Switzerland

**Keywords:** KIDSCREEN-10, Rasch model, Psychometric evaluation

## Abstract

**Aim:**

To test the psychometric properties of the KIDSCREEN-10.

**Background:**

It is important to assess mental health and well-being in children for an early detection of psychological problems or hidden morbidities. There is limited knowledge about the psychometric quality of the reduced version of the KIDSCREEN questionnaire with only 10 items.

**Methods:**

Analysis of psychometric properties was done by fitting Rasch models and graphical loglinear Rasch models to data collected in a study on acculturation of primary school children and their teachers in 2017.

**Results:**

The data did not fit a Rasch model but did fit a graphical loglinear Rasch model. There was local dependence for four item pairs and differential item functioning for gender and citizenship.

**Conclusions:**

The KIDSCREEN-10 provides essentially valid measurements of health-related quality of life in children if local dependency and dif ferential item functioning are taken into account. Reliability and targeting were less than satisfactory, especially for certain subgroups but reliability was adequate for most groups.

## Introduction

There has been a growing interest in assessing health and well-being in children and adolescents in the last decades. Early identification of mental health problems and lower subjective well-being of children and adolescents at risk for psychological problems or with hidden morbidity is important for appropriate early interventions. At least 30 instruments for measuring generic health-related quality of life (HRQoL) are available for use with children [1]. KIDSCREEN is among the instruments receiving the most attention. The questionnaires have been translated to at least 38 languages and have been used in more than 50 clinical and epidemiological studies and other research projects. KIDSCREEN is designed to assess mental health and well-being in children and adolescents aged from 8 to 18 years [2].

The KIDSCREEN project was funded by the European Commission. It took place from 2001 until 2003 and included participants of 13 European countries. The development of the questionnaires was based on literature reviews, expert discussions and children’s focus groups in all participating countries.

There are three different self-report versions with 52, 27 and 10 items, which are scored on a 5 point scale ranging from never/not at all to always/extremely. Responses were coded so that higher values indicate better HRQoL. The KIDSCREEN-52 consists of 52 items in ten dimensions: physical well-being, psychological well-being, moods and emotions, self-perception, autonomy, parent relations and home life, social support and peers, school environment, social acceptance, financial resources. The KIDSCREEN-52 has been validated with classical psychometric methods as well as Item-Response-Theory analysis and structural equation modelling [3].

The KIDSCREEN-27 is a shorter version covering the five dimensions physical well-being, psychological well-being, parent relations and autonomy, social support and peers, school environment. It has been tested similarly to the KIDSCREEN-52 [4].

The KIDSCREEN-10 covers physical well-being, moods and emotions, autonomy, family and friends as well as school environment with two items each. The time frame refers to the last week. The instrument provides a single score of HRQoL. The validity of the 10-items version is alleged to be confirmed by the same methods as the larger versions, although these results remain unpublished.

Therefore, the aim of this study was to assess the psychometric properties of the KIDSCREEN-10 using Rasch analysis. The Rasch model (RM) is an Item-Response-Theory model which was originally developed by Georg Rasch [5]. It is increasingly used in the health and psychological sciences. A Rasch analysis provides a detailed analysis of many aspects of a scale, including fit of items and persons, item bias, internal consistency, dimensionality and targeting.

## Methods

### Participants

The German version of the KIDSCREEN-10 was used in a longitudinal study about the association between acculturation orientation of immigrant students and their well-being at school and educational achievement. Parents received an information letter explaining the study. Children whose parents did not give consent, did not participate. The sample consisted of 1110 sixth-graders (580 females, 530 males) in 60 school classes from eight German speaking federal states in Switzerland. Participating school classes had to have at least 30% pupils with a migration background. The children were between 10 and 13 years old and the mean age was 12.04 years (sd = 0.54).

The data were collected during two regular consecutive classroom sessions. In the first session, students completed a questionnaire including the scales concerning their school adjustment, their psychosocial well-being, their relationship to the teacher and the KIDSCREEN-10. The children also took a literacy test and answered sociodemographic questions including gender, educational resources (number of books at home), countries of birth of themselves and their parents and citizenship. Response categories for number of books were 0–10, 11–50, 51–100 or more than 100 books. Based on their own and their parents’ country of birth the children were classified as having a migration background or not.

During the second lesson, students completed a questionnaire concerning acculturation orientation and (bi-)national identification. Children could withdraw from the study or skip parts of the questionnaire at any time. Design and results of this study are described in detail in another publication [6].

### Statistical methods

The responses to the 10 items of the KIDSCREEN-10 were first analyzed for fit to the Rasch model. The model used in this study is the partial credit model for polytomous items [7]. The overall fit of the model was tested using Andersen’s conditional likelihood ratio (CLR) test [8]. The fit of individual items was assessed by conditional outfits and infits as well as by comparing the observed item-rest-score correlations with the expected item-rest-score correlations under the model [9].

Rasch analysis is also concerned with checking differential item functioning (DIF) and local dependency (LD). Differential item functioning occurs when subgroups within the sample respond differently to an individual item despite equal levels of HRQoL. The person covariates checked for DIF were gender, educational resources, migration background and citizenship. Conditional likelihood ratio tests were conducted as overall tests for DIF [8]. Significance level was set to 5% with an adjustment for multiple testing by the Benjamini-Hochberg procedure [10].

Rasch models assume locally independent items. This means that there should be no substantial correlation left between items once the underlying latent variable has been taken into account. This assumption can be violated because the scale is not unidimensional or because of response dependence of closely related items. The assumptions of local independence and lack of DIF for individual items were tested again by conditional likelihood ratio tests [11] and by analyzing partial Goodman-Kruskal gamma coefficients [9].

Measurement quality is assessed by reliability and targeting. Cronbach’s alpha is usually calculated as a reliability estimate. It gives a lower limit to the true unknown reliability, but only if the items are locally independent. If this assumption is violated the Monte Carlo method proposed by Hamon and Mesbah [12] can be used to provide unbiased estimates of the true reliabilities. They are calculated separately for each group defined by values of the variables with DIF effects or an effect on the latent variable to take into account different variances in these groups. Hamon and Mesbah reliabilities as well as Cronbach’s alpha are reported for comparison. Targeting is the extent to which items match the study population. This was examined by comparing the distribution of item and person locations in item maps, and by the target index which is defined as the percentage of the maximum obtainable test information achieved by the mean test information. Targeting has also to be assessed separatly within each group defined by the values of the variables with DIF effects or direct effects on the latent variable.

In case of local dependence and/or DIF, extra terms to model these deviations from a Rasch model were included. This extension is known as the graphical loglinear Rasch model (GLLRM) [13]. The analysis for Rasch models and GLLRMs was carried out with DIGRAM 3.66 [14].

## Results

The overall test-of-fit (CLR test) of the Rasch model rejected item homogeneity by comparing low and high score groups, and showed DIF for all exogenous variables studied (Table 1).

**Table 1:** Conditional likelihood ratio tests for RM

Test criterion	CLR	df	$\hbox {p}^{\mathrm{a}}$
Low and high score groups	100.1	39	0.000
Gender	116.4	39	0.000
Educational resources	228.7	156	0.000
Citizenship	148.7	78	0.000
Migration	340.8	234	0.000

${}^{\mathrm{a}}$ Reject if p value $\le 0.05$

The analysis further suggested clear misfit for items (1) and (3) because there were significant differences between the observed and the expected item-rest-score correlations and because of significant outfit and infit statistics (Tables 2, 3).

**Table 2:** Item-restscore correlations for RM

Item		Observed	Expected	sd	$\hbox { p}^{\mathrm{a}}$
1	Have you felt fit and well?	0.598	0.486	0.024	0.0000
2	Have you felt full of energy?	0.553	0.477	0.026	0.0030
3	Have you felt sad?	0.385	0.484	0.025	0.0001
4	Have you felt lonely?	0.475	0.490	0.030	0.6217
5	Have you had enough time for yourself?	0.437	0.492	0.024	0.0239
6	Have you been able to do the things				
	You want to do in your free time?	0.479	0.496	0.024	0.4711
7	Have your parent(s) treated you fairly?	0.458	0.476	0.035	0.6090
8	Have you had fun with your friends?	0.475	0.474	0.028	0.9615
9	Have you got on well at school?	0.551	0.476	0.026	0.0036
10	Have you been able to pay attention?	0.470	0.469	0.027	0.9779

${}^{\mathrm{a}}$ Reject if p value $\le 0.015$

**Table 3:** Conditional outfits and infits for RM

Item	Outfit	sd	$\hbox {p}^{\mathrm{a}}$	Infit	sd	$\hbox {p}^{\mathrm{a}}$
1	0.849	0.045	0.0008	0.853	0.048	0.0020
2	0.915	0.044	0.0525	0.933	0.046	0.1449
3	1.262	0.049	0.0000	1.133	0.046	0.0038
4	1.037	0.093	0.6912	1.021	0.061	0.7301
5	1.082	0.046	0.0730	1.102	0.046	0.0253
6	1.055	0.047	0.2476	1.044	0.045	0.3299
7	1.101	0.110	0.3572	1.115	0.078	0.1411
8	0.962	0.069	0.5815	0.995	0.058	0.9270
9	0.877	0.047	0.0083	0.885	0.049	0.0193
10	0.986	0.044	0.7538	0.996	0.046	0.9223

${}^{\mathrm{a}}$ Reject if p value $\le 0.015$

The observed correlation between item (3) (“Have you felt sad?”) and the remaining items was much weaker than expected. Obviously, even children who feel well in general can feel sad from time to time. Item (1) (“Have you felt fit and well?”) had a higher correlation with the other items than expected, it appears to be a global indicator for well-being. Deleting item (3) hardly improved the fit. The CLR test still rejected the Rasch model and there was a lot of DIF and evidence of local dependence between pairs of items, which didn’t come as a surprise, as the items had been chosen pairwise from different dimensions.

**Fig. 1:**
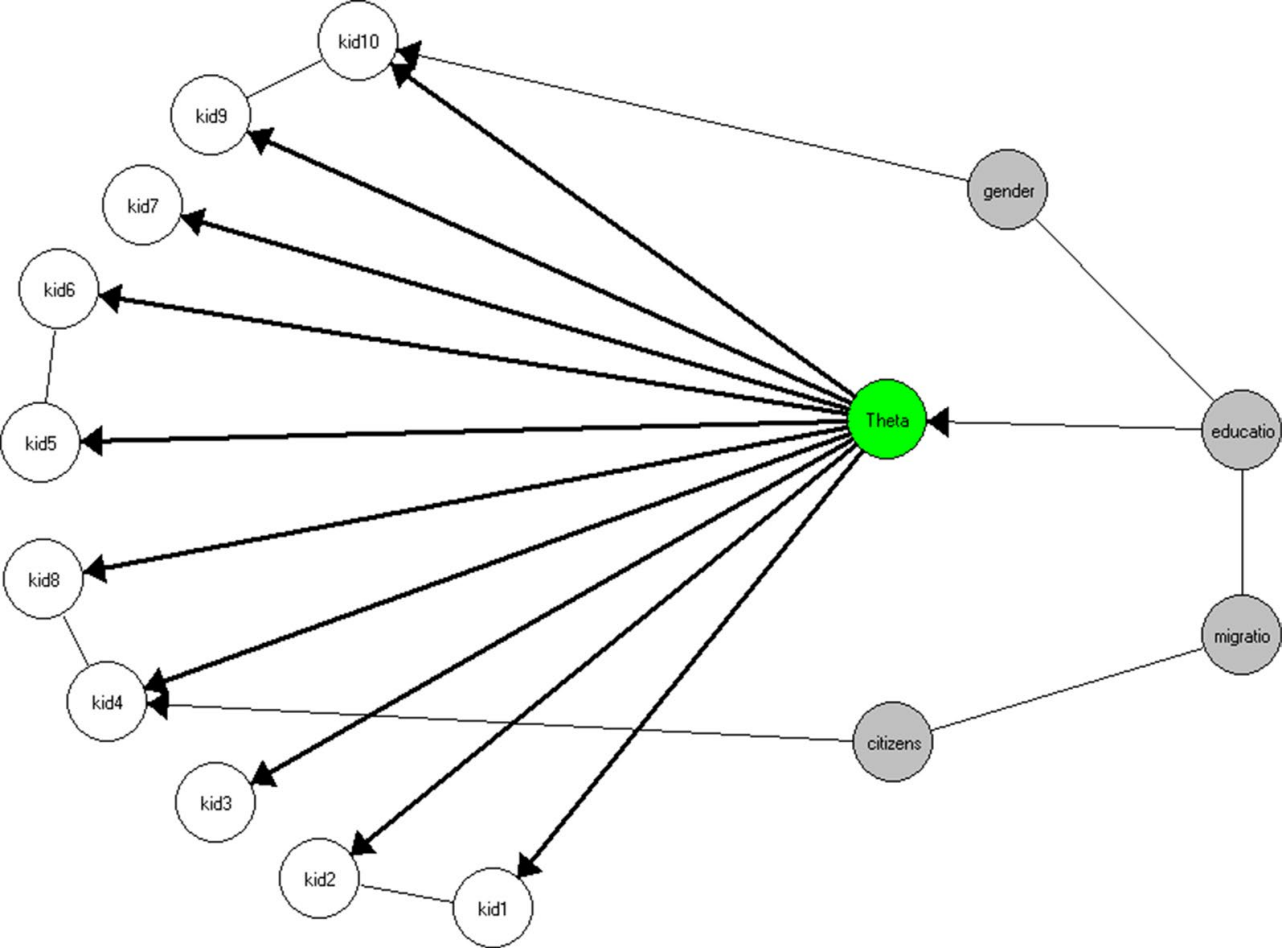
Graphical representation of the GLLRM model

Fit to the graphical loglinear Rasch model (GLLRM) was analyzed next. The Item Response Theory graph in Fig. 1 shows the relationships between the items, the latent variable and exogenous covariables. A missing connection between two nodes indicates that the variables are conditionally independent, given the other variables in the model. An undirected edge between two nodes indicates that the variables are conditionally dependent without assuming a causal relationship, an arrow indicates a causal relationship. The graph shows that there is LD between items 1 and 2 (physical well-being), items 4 and 8 (social well-being), items 5 and 6 (autonomy) as well as items 9 and 10 (school). These item pairs come from different dimensions of the original KIDSCREEN-52. The graph also indicates two DIF effects. Gender has a DIF effect on item 10 and citizenship has a DIF effect on item 4. Examining the subgroups reveals that girls score higher on the item about attention at school then boys given the same value of HRQoL. Children with a double citizenship are more likely to endorse the item about loneliness than children with another citizenship, and these are more likely to feel lonely than children with Swiss citizenship. There is a direct effect of educational resources on HRQoL.

The overall fit of the GLLRM and item fit statistics are examined as for the Rasch model before. The CLR tests in Table 4 show item homogeneity ($p=0.332$) and lack of DIF for educational sources ($p=0.064$) and migration background ($p=0.403$). DIF by gender and citizenship is confirmed by significant test results.

**Table 4:** Overall CLR tests for GLLRM

Test criterion	CLR	df	$\hbox {p}^{\mathrm{a}}$
Low and high score groups	115.9	110	0.332
Gender	158.9	103	0.000
Educational resources	486.0	440	0.064
Citizenship	254.9	196	0.003
Migration	668.3	660	0.403

${}^{\mathrm{a}}$ Reject if p value $\le 0.0125$

The tests of local dependence and DIF for single items are shown in Table 5. All tests are clearly significant and reflect what can be seen in Fig. 1.

**Table 5:** Specific CLR tests for GLLRM

Type	Item	Item/covariable	CLR	df	p
LD	kid1	kid2	232.3	16	0.0000
LD	kid4	kid8	42.53	16	0.0003
LD	kid5	kid6	55.33	16	0.0000
LD	kid9	kid10	142.85	16	0.0000
DIF	kid4	citizenship	29.41	8	0.0003
DIF	kid10	gender	35.41	4	0.0000

**Table 6:** Item-restscore correlations for GLLRM

Item	Observed	Expected	se	$\hbox {p}^{\mathrm{a}}$
1	0.598	0.546	0.023	0.0242
2	0.553	0.555	0.023	0.9252
3	0.385	0.412	0.026	0.2924
4	0.475	0.465	0.031	0.7402
5	0.437	0.477	0.025	0.1111
6	0.479	0.485	0.024	0.8189
7	0.458	0.401	0.037	0.1222
8	0.475	0.458	0.029	0.5543
9	0.551	0.498	0.025	0.0372
10	0.470	0.498	0.026	0.2763

$^{\mathrm{a}}$ Reject if p value $\le 0.00147$

All outfit and infit statistics show good fit to the model and the differences between the observed and the expected item-rest-score correlations were not significant when taking into account an adjustment for multiple testing (see Tables 6, 7).

**Table 7:** Conditional outfits and infits for GLLRM

Item	Outfit	se	$\hbox {p}^{\mathrm{a}}$	Infit	se	$\hbox {p}^{\mathrm{a}}$
1	0.935	0.048	0.1700	0.924	0.049	0.1224
2	1.030	0.044	0.4865	1.048	0.046	0.2969
3	1.083	0.047	0.0793	1.014	0.046	0.7578
4	0.988	0.087	0.8908	0.992	0.062	0.8961
5	1.060	0.045	0.1883	1.084	0.046	0.0670
6	1.023	0.048	0.6320	1.024	0.046	0.6032
7	0.933	0.101	0.5078	0.997	0.081	0.9707
8	0.919	0.067	0.2297	0.973	0.060	0.6562
9	0.914	0.046	0.0606	0.918	0.048	0.0889
10	1.036	0.042	0.3969	1.046	0.044	0.2950

$^{\mathrm{a}}$ Reject if p value $\le 0.00147$

**Fig. 2:**
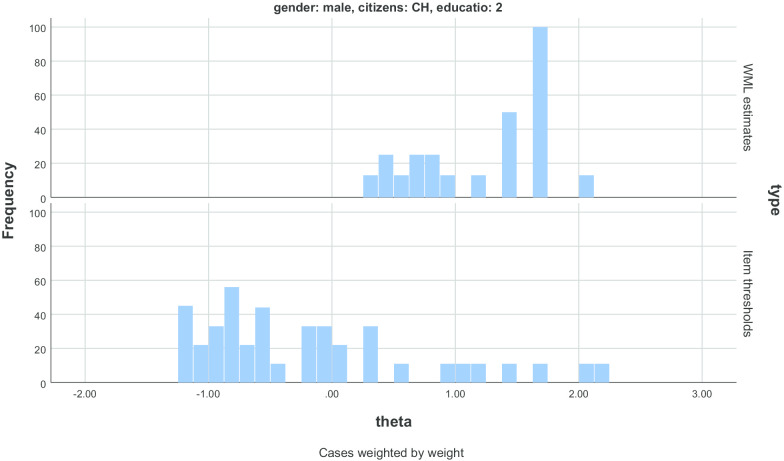
Item map for Swiss boys with little educational resources

Cronbach’s alpha for the ten items was 0.79. The true reliability for swiss boys with little educational resources (not more than 50 books at home) was 0.38. For girls with a double or another citizenship and little educational resources, the true reliability also was rather low with 0.56 and 0.57. For all other groups, reliabilities were between 0.66 and 0.85. The average amount of test information was only 42% of the possible information required for perfect targeting for swiss boys with little educational resources. Targeting was slighly better for the other groups with percentages up to 67% of the maximum obtainable information. The item map in Fig. 2 shows the distributions of person estimates for Swiss boys with little eductional resources in the upper part of the plot and the item thresholds in the lower part of the plot. The distribution of these children is on the right hand side of the distribution of item thresholds with little overlapping. So, the KIDSCREEN targets children with lower HRQoL than that found in this subgroup.

The standard error of measurement (SEM) of the total scores is another indicator for measurement quality. The subgroups have average SEM values between 2.19 and 2.90. This means that the scores are not very precise, in accordance with the suboptimal targeting.

Finally, DIF equated scores were calculated to assess the impact of DIF. Differences between observed and adjusted scores were rather small (less than 1.5) compared to the measurement error. The average scores for the subgroups with and without adjustment are given in Table 8. The bias is equal to the difference between the two mean values. The adjustment for DIF regarding citizenship has practically no effect, differences between group means are unchanged. The difference between unadjusted means of girls and boys is 0.44, which goes up to 0.76 with DIF adjustment. Hence, with the adjustment boys have on the average substantially higher scores than girls.

**Table 8:** Observed and adjusted scores accounting for DIF

	Observed		Adjusted		Bias
	Mean	se	Mean	se	
Gender					
Girls	31.57	0.21	31.50	0.21	0.07
Boys	32.01	0.23	32.26	0.22	− 0.24
Citizenship					
Swiss	32.39	0.26	32.52	0.26	− 0.13
Swiss and another	31.69	0.26	31.64	0.26	0.05
Another	31.34	0.27	31.50	0.26	− 0.16

## Discussion

The data did not fit a Rasch model. The CLR test rejected item homogeneity, there were two misfitting items, (1) and (3), and a number of items showed local dependence and DIF. We have found only one other study that evaluated the psychometric properties of the KIDSCREEN-10 using Rasch modelling [15] . They did not examine local dependence or DIF, they only reported infit mean squares for the items. Interestingly, item (3) (“Have you felt sad?”) was also misfitting there, whereas item (1) (“Have you felt fit and well?”) caused no problems.

Overall the KIDSCREEN-10 demonstrated good validity and reliability when a GLLRM was fitted to allow for local dependence among items and DIF. Several item pairs exhibited local dependence as they belonged to different dimensions of the original 52-items instrument. This is not a big problem for producing valid scores for HRQoL because the local dependence can be taken into account by collapsing the item pairs to a superitem with possible scores between 0 and 8, instead of scores between 0 and 4. However, since several pairs of items are locally dependent, Cronbach’s alpha gives an overestimate of the true reliability.

The DIF analysis showed that the answer to item 10 about school attention varied for girls and boys, and the answers to item 4 about feeling lonely were different depending on the citizenship of children, given the same level of HRQoL. To compare total scores among children of different sex or citizenship, appropriate adjustments have to be made. As the sample size is large small model deviations will be significant. Hence, there might be significant but not relevant DIF effects. Of the two DIF variables only gender seems to matter. Because girls have fewer problems with school attention than boys the girls’ scores seem to be similar to the scores of the boys. If this difference in school attention is taken into account the difference in total scores between boys and girls becomes more evident. Gender differences in well-being have been found in a lot of studies. If children participated they mostly were in adolescence or even young adults. Since it is also known that the gender gap increases with age it is important to investigate the target age group and carefully adress any DIF effects.

Targeting in this study was not very good. The distribution of participants shows higher values than the distribution of item thresholds. Estimates of person parameters are therefore imprecise, which corresponds to the size of the standard error measurement. Reliability was adequate for all but two subgroups.

### Strengths and limitations

The KIDSCREEN-10 is a shortened version of a carefully designed and well-validated questionnaire to measure health-related quality of life of children and adolescents. The KIDSCREEN-52 has been widely used in a lot of different countries. This examination of the KIDSCREEN-10 meets all the requirements of a high quality psychometric validation. Furthermore, the sample size is quite large. On the other hand, the results cannot so easily be generalized. The children in this study come from schools that are very conscious of migration issues and equal opportunities. It can be assumed that the children are taught in a supportive environment, and they are also quite ambitious. These factors, together with the data collection process during classroom sessions, could well lead to more positive answers than expected.

## Conclusions

The KIDSCREEN-10 provides essentially valid measurements of HRQoL in children if local dependency and DIF are taken into account. Reliability and targeting were less than satisfactory, especially for certain subgroups. The applicability of this instrument to other target groups should be further investigated.

## Data Availability

The datasets generated and analyzed during the current study are available from the corresponding author on reasonable request.

## Abbreviations

CLR: Conditional likelihood ratio
DIF: Differential item functioning
GLLRM: Graphical loglinear Rasch model
HRQoL: Health-related quality of life
LD: Local dependence
RM: Rasch model
